# Interactions between mobile genetic elements: An anti-phage gene in an integrative and conjugative element protects host cells from predation by a temperate bacteriophage

**DOI:** 10.1371/journal.pgen.1010065

**Published:** 2022-02-14

**Authors:** Christopher M. Johnson, M. Michael Harden, Alan D. Grossman

**Affiliations:** Department of Biology, Massachusetts Institute of Technology, Cambridge, Massachusetts, United States of America; Université de Sherbrooke: Universite de Sherbrooke, CANADA

## Abstract

Most bacterial genomes contain horizontally acquired and transmissible mobile genetic elements, including temperate bacteriophages and integrative and conjugative elements. Little is known about how these elements interact and co-evolved as parts of their host genomes. In many cases, it is not known what advantages, if any, these elements provide to their bacterial hosts. Most strains of *Bacillus subtilis* contain the temperate phage SPß and the integrative and conjugative element ICE*Bs1*. Here we show that the presence of ICE*Bs1* in cells protects populations of *B*. *subtilis* from predation by SPß, likely providing selective pressure for the maintenance of ICE*Bs1* in *B*. *subtilis*. A single gene in ICE*Bs1* (*yddK*, now called *spbK* for SPß killing) was both necessary and sufficient for this protection. *spbK* inhibited production of SPß, during both activation of a lysogen and following *de novo* infection. We found that expression *spbK*, together with the SPß gene *yonE* constitutes an abortive infection system that leads to cell death. *spbK* encodes a TIR (Toll-interleukin-1 receptor)-domain protein with similarity to some plant antiviral proteins and animal innate immune signaling proteins. We postulate that many uncharacterized cargo genes in ICEs may confer selective advantage to cells by protecting against other mobile elements.

## Author summary

Chromosomes from virtually all organisms contain genes that were horizontally acquired. In bacteria, many of the horizontally acquired genes are located in mobile genetic elements, elements that promote their own transfer from one cell to another. These elements include viruses and conjugative elements that are parts of the host genome and they can contain genes involved in metabolism, pathogenesis, symbiosis, and antibiotic resistances. Interactions between these elements are poorly understood. Furthermore, the majority of these elements confer no obvious benefit to host cells. We found that the presence of an integrative and conjugative element (ICE) in a bacterial genome protects host cells from predation by a bacteriophage (virus). There is a single gene in the integrative and conjugative element that confers this protection, and one gene in the bacteriophage that likely works together with the ICE gene. When expressed at the same time, these two genes cause cell death, before functional viruses can be made and released to kill other cells. We postulate that other ICEs may confer selective advantage to their host cells by protecting against other mobile elements.

## Introduction

Mobile genetic elements can move between host genomes or within a host’s genome. The genomes of many bacterial species contain multiple functional and defective mobile elements, including insertion sequences, transposons, temperate phages, genomic islands, and integrative and conjugative elements (ICEs; also called conjugative transposons). In some cases, these elements constitute a substantial portion of the host genome [1–4]. Multiple elements within a given host have the potential to interact with each other, and likely co-evolve.

ICEs are mobile genetic elements that reside integrated in a host chromosome and are replicated and segregated to daughter cells along with the host genome [5–7]. Under certain conditions, or stochastically, an ICE can excise from the chromosome and be transferred to a recipient cell via the element-encoded conjugation machinery, typically a type IV secretion system.

ICEs frequently carry cargo genes that are not essential for their own lifecycle, but instead benefit the host. Most well-studied ICEs were discovered because of such phenotypes [6]. For example, the ICE Tn*916* was discovered because it confers tetracycline resistance to host cells and can move between cells via conjugation [8,9]. Likewise, many other ICEs were identified because they carry genes that confer specific phenotypes including: antibiotic resistance [10–15], pathogenesis [16], symbiosis [17], and metabolic functions [18–21].

Many ICEs have been identified by means other than the phenotype conferred by their cargo genes. In these cases, the functions of the cargo genes are largely unknown. We suspect that many of these cargo genes encode functions that are beneficial to the host under certain conditions, but that the appropriate conditions have not been identified.

Many strains of *Bacillus subtilis* contain at least two functional mobile genetic elements, the integrative and conjugative element ICE*Bs1* [22,23] and the temperate phage SPß [24]. *B*. *subtilis* strains also contain several defective mobile genetic elements [25–27].

**Fig 1:**
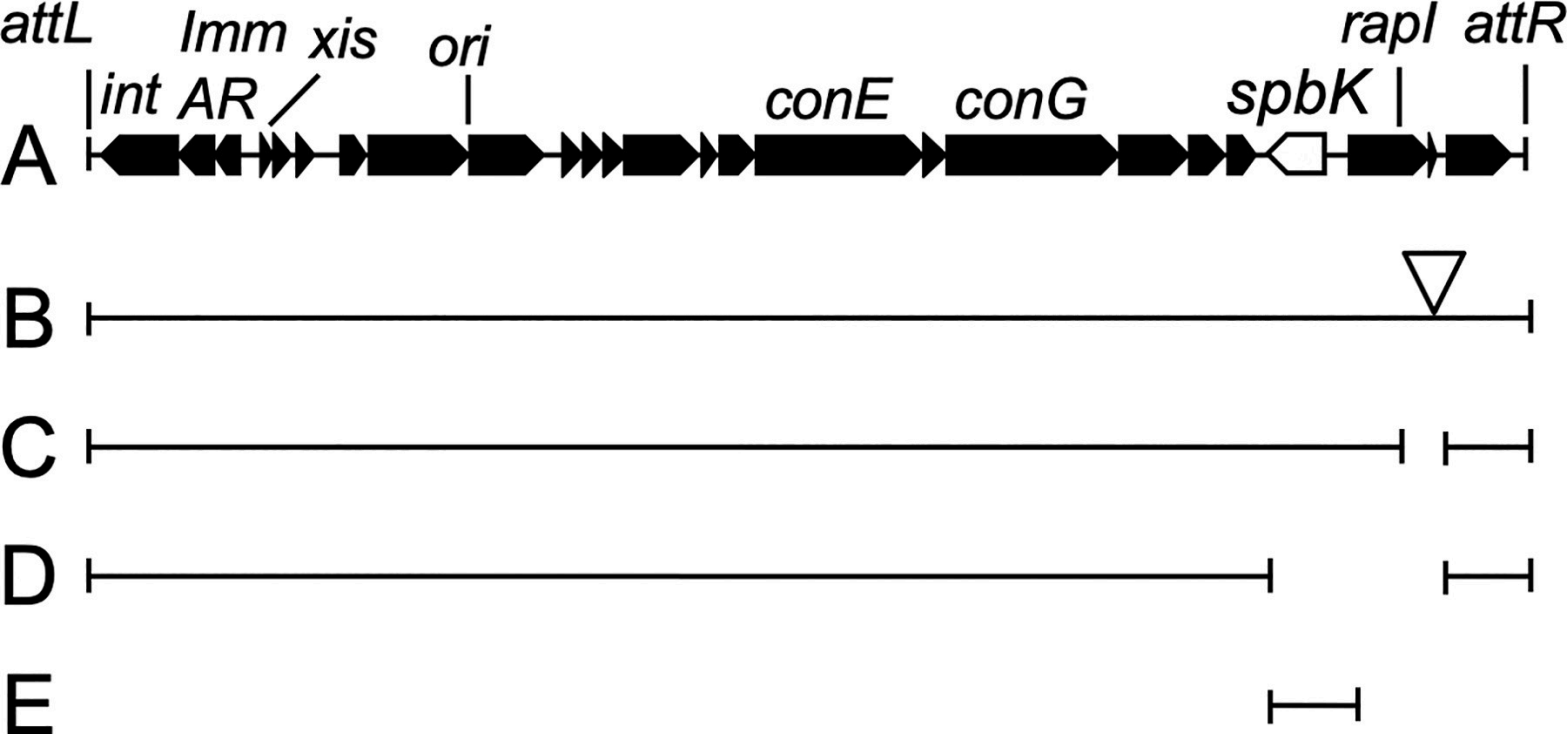
Map of ICE*Bs1* and some mutants. **A.** A linear map of ICE*Bs1* is shown. Genes are indicated as filled boxes with arrowheads at the ends indicating the direction of transcription. *spbK* is shown as an open arrow. The attachment sites *attL* and *attR* mark the junctions between ICE*Bs1* and chromosomal sequences. Below the map are shown the ICE*Bs1* mutants used in this work. The regions of ICE*Bs1* that are present in each construct are shown as bars beneath the map. **B.** ICE*Bs1*::*kan*. The open triangle indicates that this construct contains a *kan* gene inserted in the intergenic region between *rapI* and *yddM*. **C.** ICE*Bs1* Δ(*rapI-phrI*). **D.** ICE*Bs1* Δ(*spbK-phrI*). **E.** ICE*Bs1*^0^ *spbK*+. Many of these derivatives of ICE*Bs1* are used in multiple strains as indicated in Table 1.

ICE*Bs1* (Fig 1) is found integrated in the *B*. *subtilis* genome in *trnS-leu2*, the gene for a leucine-tRNA. While integrated, most ICE*Bs1* genes are repressed [22,28]. ICE*Bs1* is activated during the *recA-*dependent SOS response to DNA damage or in the presence of *B*. *subtilis* cells that do not have the element [22]. Under these conditions, ICE*Bs1* gene expression is derepressed, the element excises from the chromosome and can transfer to an available recipient via the element-encoded conjugation machinery. ICE*Bs1* was identified because of homology to other ICEs [29] and because it is regulated by cell-cell signaling [22]. At the time of its discovery, it was not known if ICE*Bs1* conferred a beneficial phenotype to its host.

SPß is a temperate phage found in the chromosome of many isolates of *B*. *subtilis*. Historically, SPß was thought to be a defective phage {reviewed in [30]}. When strains cured of SPß (SPß^0^) were isolated, it became clear that it is functional [31], and cured strains are used to grow the phage. A widely used strain missing SPß is also missing ICE*Bs1* [32,33].

In strains lysogenic for SPß, the phage is integrated in *spsM*, near the terminus of replication [32,34,35]. SPß contains genes needed for production of and resistance to the peptide antibiotic sublancin [35,36], providing a growth advantage to the host in the presence of cells sensitive to sublancin. Most phage genes are repressed in the lysogen, but during the *recA*-dependent SOS response to DNA damage, SPß gene expression is induced and the phage excises from the host chromosome. In cells capable of producing phage particles, the activated phage enters the lytic cycle, produces progeny phage, and causes cell lysis and release of phage particles.

We found that the presence of ICE*Bs1* in *B*. *subtilis* inhibited production of active SPß, both when the phage was activated from the lysogenic state and during *de novo* infection. The ICE*Bs1* gene *spbK*, although dispensable for conjugation, was necessary and sufficient for the inhibition of SPß. The *spbK* gene product contains a Toll/Interleukin-1 Receptor (TIR) domain that was needed for function. The anti-SPß phenotype (abortive infection) caused by *spbK* was dependent on the SPß gene *yonE*. We found that *yonE* was essential for SPß lytic growth, but not for establishing a lysogen. Co-expression of *spbK* and *yonE* inhibited host cell growth and caused a drop in cell viability, even in the absence of any other ICE*Bs1* or SPß genes. The presence of ICE*Bs1* in cells prevented the spread of SPß, thereby protecting nearby *B*. *subtilis* cells from infection and allowing the population to continue growing. This phenotype likely provides strong selective pressure to maintain ICE*Bs1* in *B*. *subtilis*. We postulate that other ICEs might encode abortive infection, or other anti-phage systems, providing selective pressure for host cells to maintain these ICEs.

## Results

### ICE*Bs1* prevents SPß from forming plaques

**Fig 2:**
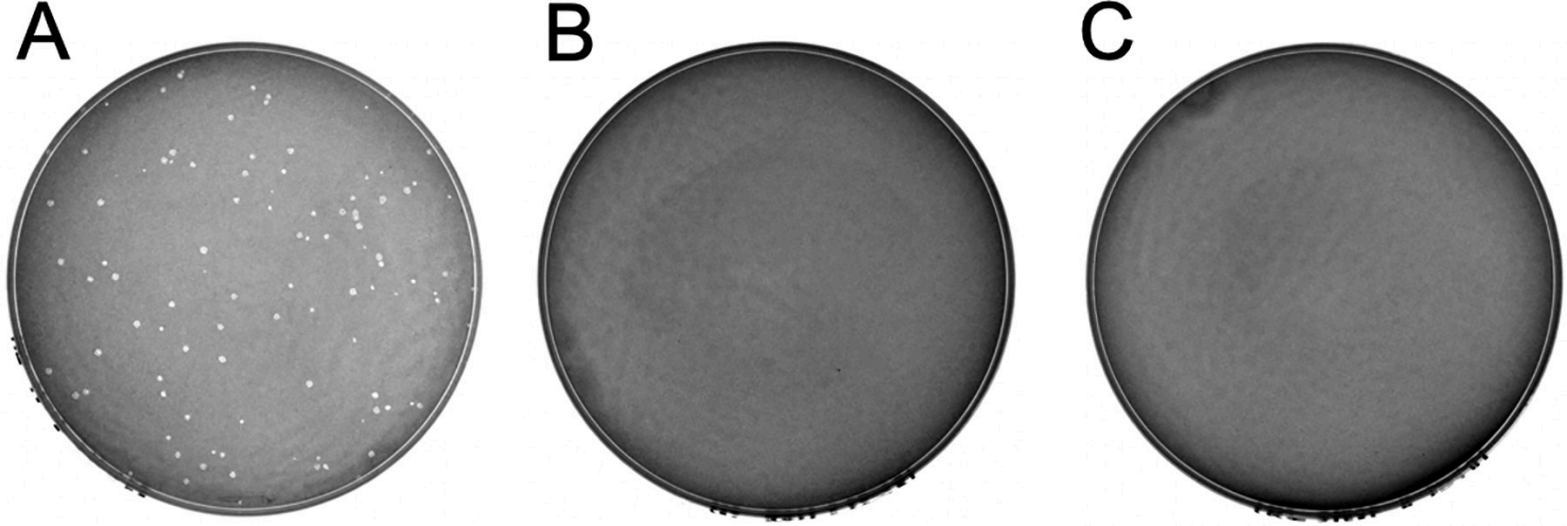
ICE*Bs1* prevents plaque formation by SPß. Various numbers of PFUs of SPß were mixed with the indicated strains, plated, incubated overnight and checked for the presence of plaques (methods). **A.** Approximately 100 PFUs of SPß were mixed with the indicator strain CU1050. **B.** Approximately 100 PFUs of SPß mixed with strain CMJ81 (the indicator strain CU1050 carrying ICE*Bs1*). **C.** Approximately 10^4^ PFU of SPß mixed with strain CMJ81 (the indicator strain CU1050 carrying ICE*Bs1*).

ICE*Bs1* was not known to confer phenotypes to *B*. *subtilis*, aside from those directly related to conjugation. However, the left end of ICE*Bs1* (Fig 1) encodes a phage-like repressor ImmR [28], anti-repressor ImmA [37], and recombinase Int [38]. In addition, the strain CU1050, that is cured of and often used to grow the temperate subtilis phage SPß [30,31], still contains the defective prophage PBSX and *skin* but is cured of ICE*Bs1* [33]. This information led us to wonder if there might be some interaction between ICE*Bs1* and SPß. We tested if the presence of ICE*Bs1* in *B*. *subtilis* altered the ability of SPß to make plaques. We mixed SPß with two *B*. *subtilis* strains, one that was missing ICE*Bs1* (ICE*Bs1*^0^) and that has been used as an indicator strain for SPß strain (CU1050) [32,33] and an isogenic derivative that contained ICE*Bs1* (CMJ81; ICE*Bs1*+). SPß formed plaques on a lawn of the ICE*Bs1*^0^ strain (Fig 2A), but not on a lawn of the isogenic ICE*Bs1+* strain (Fig 2B), even when 100-fold more plaque forming units (PFUs) were mixed with cells (Fig 2C). Based on these results, we conclude that the presence of ICE*Bs1* inhibited plaque formation by SPß.

### ICE*Bs1* reduces phage production during infection

**Fig 3:**
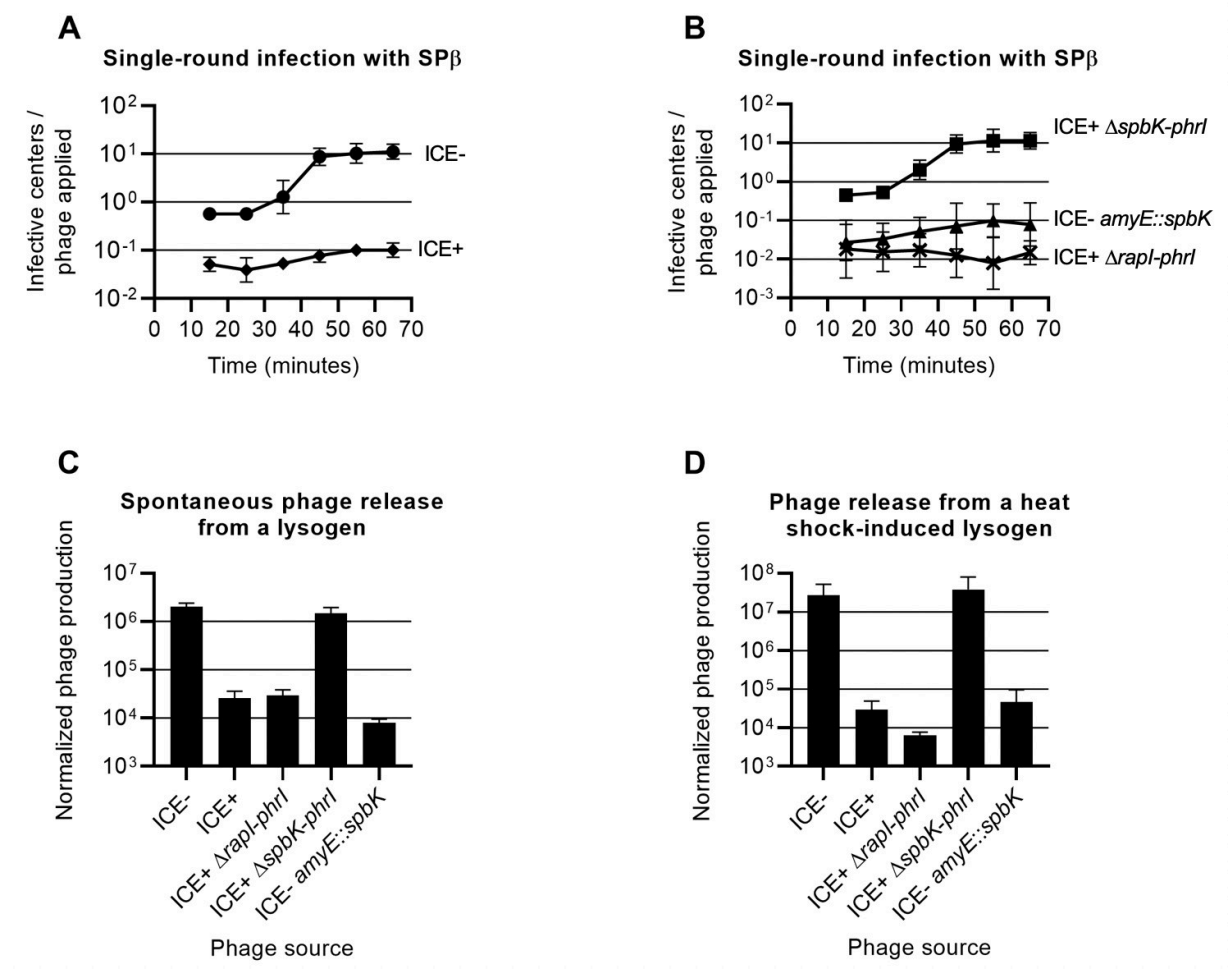
Production of SPß during the lytic cycle is reduced in cells containing ICE*Bs1* or *spbK*. **A.** Effect of ICE*Bs1* on single-round infection of *B*. *subtilis* cultures. SPß null strains were grown in rich medium, infected with phage (MOI = 0.01) and then diluted in fresh medium. The number of infective centers in the culture was tracked over time using strain CU1050 as the indicator (methods). circles, ICE*Bs1*^0^ (CU1050); diamonds, ICE*Bs1*^+^ (CMJ81). **B.** Effect of *spbK* on single-round infection of *B*. *subtilis* cultures. SPß null strains were grown and infected with SPß as in 2A. crosses, ICE*Bs1*^+^ Δ*rapI-phrI* (CMJ913); squares, ICE*Bs1*^+^ Δ(*spbK-rapI-phrI*) *(*CMJ914); triangles, ICE*Bs1*^0^ *amyE*::*spbK* (CMJ82). **C.** Effect of ICE*Bs1* and *spbK* on spontaneous phage production. Strains carrying wild type SPß lysogens and different ICE*Bs1* variants were grown in rich medium; ICE*Bs1*^0^ (JMA222), ICE*Bs1*^+^ (AG174), ICE*Bs1*^+^ Δ*rapI-phrI* (IRN342), ICE*Bs1*^+^ Δ(*spbK-rapI-phrI*) *(*CAL1500), ICE*Bs1*^0^ *amyE*::*spbK* (CMJ74). Supernatant was collected from each culture during exponential growth and used as a phage source in a plaque assay (methods). **D.** Effect of ICE*Bs1* and *spbK* on phage production after induction of a lysogen. Strains carrying ts SPß lysogens and different ICE*Bs1* variants were grown in rich medium; ICE*Bs1*^0^ (CMJ114), ICE*Bs1*^+^ (CMJ826), ICE*Bs1*^+^ Δ*rapI-phrI* (CMJ917), ICE*Bs1*^+^ *Δ(spbK-rapI-phrI*) (CMJ918), ICE*Bs1*^0^ *amyE*::*spbK* (CMJ116). SPß lysogens were induced by a heat shock during exponential growth and supernatants were collected and used as a phage source in a plaque assay (methods). For C and D the Y axis shows the number of PFU/ml of culture divided by the OD600 of the culture.

To quantify the effects of ICE*Bs1* on the production of SPß, we measured the kinetics of phage production during a single round of infection (Fig 3A and 3B). We mixed ~10^5^ PFUs of SPß with ~10^7^ cells (MOI ~0.01) for 5 min at 37°C, pelleted the cells by centrifugation, washed the cells to remove unattached phage, and resuspended the cells in LB medium at 37°C to allow for phage growth. The initial number of infective cells in the medium was determined by measuring the number of infective centers (PFUs) following the initial adsorption, and new phage production was monitored by tracking the subsequent increase in infective centers. For a strain without ICE*Bs1*, the initial number of infective centers in the culture was about 90% of the initial number of phage used to inoculate the culture (Fig 3A). The number of infective centers in the culture began to increase about 25 minutes after initial infection, and plateaued about 45 minutes after initial infection. This indicated that SPß had an eclipse period of about 25 minutes (Fig 3A). The burst size (number of phage produced per infective cell) was 20 ± 7, somewhat less than the previously reported burst size of about 30 phage [24].

Cells with ICE*Bs1* that were exposed to SPß were less likely to become infective centers, and produced fewer phage per initial infective center. At an MOI of 0.01, the number of cells that produced any phage was reduced at least 10-fold relative to cells without ICE*Bs1* (Fig 3A). Furthermore, the number of phage produced per initial infective center was ~2.2 ± 0.4 (Fig 3A). Based on these results, we conclude that the presence of ICE*Bs1* in cells reduced the total number of phage released from the infected culture by at least 100-fold, or to about 0.1 progeny phage per infecting phage. This reduction did not support propagation of phage in the lytic cycle.

### ICE*Bs1* has little or no effect on entry of phage into cells

The ICE*Bs1*-dependent reduction in plaque formation and phage production could be due to reduced entry of phage into cells. Alternatively, a step in the phage lifecycle after entry could be inhibited. If the presence of ICE*Bs1* was causing a block in phage entry, then there should be a corresponding reduction in the frequency of lysogen formation. We used SPß that contained *spc*, conferring resistance to spectinomycin, to measure the frequency of lysogenization. Cells with or without ICE*Bs1* were mixed with SPß::*spc98* (MOI ~ 0.001), unbound phage were washed off, and cells were spread on plates containing spectinomycin to select for lysogens. The lysogenization frequency of cells without ICE*Bs1* (CMJ472) was ~1% (1.1x10^-2^ ± 0.46x10^-2^), or approximately one lysogen per 100 initial phage. Similarly, the lysogenization frequency of ICE*Bs1*+ cells (CMJ827) was ~0.4% (4.3x10^-3^±1.3x10^-3^), or about 40% of that of the ICE*Bs1*^0^ cells. These results indicate that ICE*Bs1* has a relatively minor (if any) effect on lysogenization frequency and that the anti-SPß phenotype conferred by ICE*Bs1* was not due to a block in adhesion or entry of the phage.

### ICE*Bs1* reduces the number of phage released by SPß lysogens

We found that the presence of ICE*Bs1* in an SPß lysogen inhibited phage production. We grew lysogens in liquid medium and measured the number of PFUs present in the supernatant. We found that cultures of a lysogen without ICE*Bs1* had approximately 100-fold more PFUs/ml than cultures of an ICE+ lysogen (Fig 3C). Together our results demonstrate that ICE*Bs1* acts primarily by blocking production of phage by infected cells, rather than by preventing infection of cells in the first place.

We also found that the presence of ICE*Bs1* prevented production of SPß following induction of a temperature sensitive lysogen. We grew strains with a temperature sensitive SPß lysogen (SPß*c2*) in rich medium, induced the lysogen by heat shock, and measured phage release. Phage production was reduced by ~1,000-fold in cells with ICE*Bs1* compared to cells without (Fig 3D). Although production of functional phage particles was reduced, the cells were still killed following phage induction. Cell viability, as measured by colony forming units (CFUs), was reduced to ~0.1% after phage induction compared to right before phage induction for strains with (CMJ826) and without ICE*Bs1* (CMJ114). Based on these results we conclude that ICE*Bs1* was probably not preventing induction of SPß but rather was inhibiting production of active phage particles post-induction.

### The ICE*Bs1* gene *spbK* is necessary and sufficient to inhibit SPß

We were interested in determining which ICE*Bs1* gene was responsible for the inhibition of SPß. Most ICE*Bs1* genes are repressed when ICE*Bs1* is integrated in the host genome. Because the inhibition of SPß did not appear to depend on activation of ICE*Bs1*, we focused on the handful of ICE*Bs1* genes that are constitutively expressed, including genes toward the left and right ends of the element (Fig 1). Preliminary experiments led us to focus on *spbK* (formerly *yddK*). These experiments included testing for the presence of SPß in the culture supernatant from lysogens, essentially as described above, with various regions of ICE*Bs1* deleted. Most deletion mutants tested had been described previously [22,39–41]. The preliminary results indicated that strains in which *spbK* was intact, including Δ*cwlT* and *ΔrapI-yddM*, inhibited phage release. In contrast, strains in which *spbK* had been deleted, including Δ*conG-yddM*, Δ*ydcB-yddM*, Δ*nicK-yddM*, and *ΔydcQ-yddM* [39], did not inhibit phage production. Based on these results, we inferred that *spbK* was likely needed for ICE*Bs1*-mediated inhibition of spontaneous release of SPß from a lysogen and tested this directly. We used three different assays to test the effects of *spbK* on SPß. In all three assays, we compared three *B*. *subtilis* strains: an ICE*Bs1*+ strain with *spbK* (*Δ*(*rapI-phrI*)::*kan*, Fig 1C), an ICE*Bs1*+ strain lacking *spbK* (*Δ*(*spbK-phrI*)::*kan*, Fig 1D), and an ICE*Bs1*^0^ strain expressing *spbK* from its own promoter at an ectopic locus (ICE*Bs1*^0^ *amyE*::{*spbK kan*}, Fig 1E). We measured: 1) the appearance of infective centers following a single round of infection with SPß (Fig 3A and 3B); 2) the number of phage spontaneously released from an SPß lysogen (Fig 3C); and 3) the number of phage produced after induction of a temperature sensitive SPß lysogen (Fig 3D). In all cases, we found that *spbK* was necessary for ICE*Bs1* to inhibit the formation of infective centers and the production of phage, and that ICE*Bs1*+ *ΔspbK* strains were indistinguishable from strains entirely lacking ICE*Bs1*. Furthermore, ectopic expression of *spbK* was sufficient to inhibit phage production in the absence of ICE*Bs1* in all three assays.

### Expression of the SPß gene *yonE* inhibits acquisition of ICE*Bs1* and this inhibition is dependent on the ICE*Bs1* gene *spbK*

Based on the results described above, we thought that there might be at least one gene in SPß that was needed for the *spbK*-mediated inhibition of phage production. Results described below indicate that *yonE* is this SPß gene.

In previous work [42], we used Tn-seq to identify genes in recipients that affected the efficiency of stable acquisition of ICE*Bs1* in conjugation. Briefly, a library of random transposon insertions in a strain that is an SPß lysogen and cured of ICE*Bs1* was used as the recipient in conjugation. We selected for transconjugants that had acquired ICE*Bs1*. Insertion mutations that cause a decrease in acquisition of ICE*Bs1* were underrepresented in transconjugants relative to controls. We found that insertions in some position in the SPß gene *yonF* were underrepresented, indicating that these insertion mutations reduced the ability of would-be recipients to stably acquire ICE*Bs1* from donors. Because the frequency of insertions in other positions in *yonF* was unaltered in transconjugants [42], and because neither *yonF* nor *yonE* is normally expressed in SPß lysogens, the phenotype could not be due to loss of *yonF*. We hypothesized that the reduction in acquisition of ICE*Bs1* might be due to inappropriate expression of *yonE*, the gene immediately downstream of *yonF*, likely transcribed from the promoter for the antibiotic resistance gene (*spc*) in the transposon. We therefore tested directly the effects of inappropriate expression of *yonE* on acquisition of ICE*Bs1*.

**Fig 4:**
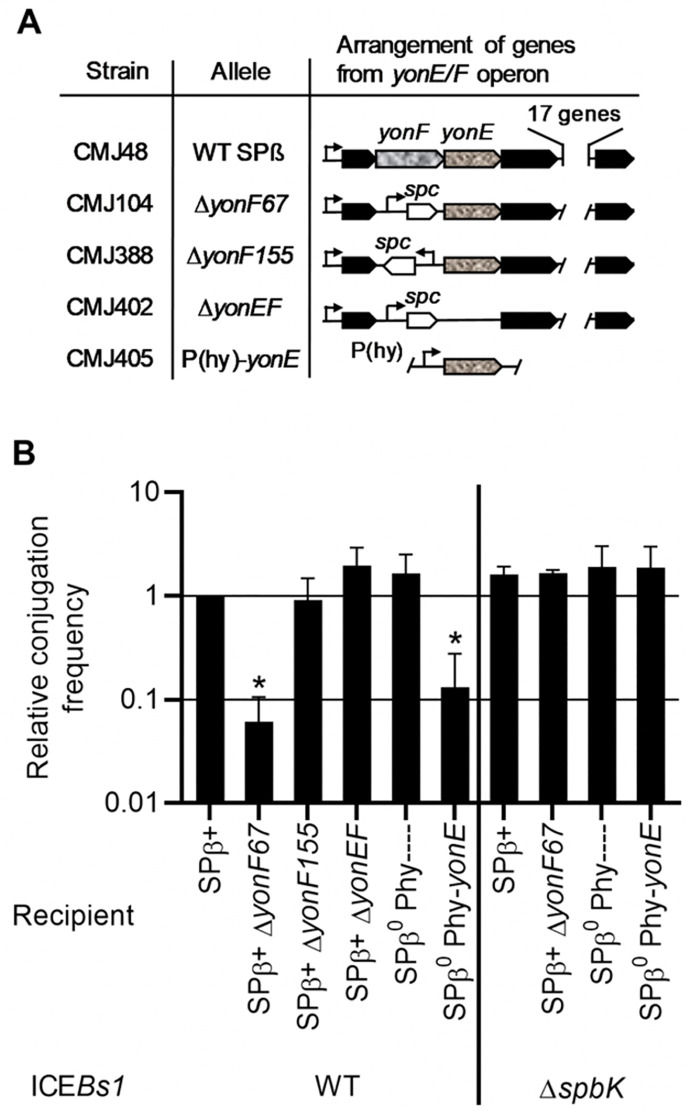
Expression of *yonE* in recipients reduces acquisition of ICE*Bs1* via conjugation. **A.** Map of the operon in SPß that contains *yonF* and *yonE*, and relevant mutations. Genes are shown as arrows. *yonF* and *yonE* are indicated by arrows filled with a mottled pattern. *spc* is shown as an open arrow. Promoters are shown as bent arrows. The allele and the recipient strain carrying that allele are indicated. **B.** The relative conjugation frequencies are shown, normalized to the conjugation frequency between a donor carrying a wild type ICE*Bs1* (KM250) and a recipient with a wild type SPß (CMJ48) within the same experiment, (average 9.7 x 10^−4^ ± 1.3 x 10^−3^ transconjugants/donor). The Δ*spbK* donor (CMJ431) carries an ICE*Bs1* in which *spbK-rapI-phrI* have been deleted. The recipient with the promoter Pspank(hy) and no *yonE* allele is CMJ405. Other recipient strain numbers are indicated in panel A. Each experiment was repeated ≥ 3 times. Asterisks indicate that the conjugation frequency with the given recipient is significantly different than that with the wild type control (p<0.05, t-test).

We found that inappropriate expression of *yonE* reduced the ability of cells to stably acquire a copy of ICE*Bs1*. We made a series of mutations in SPß (Fig 4A) and tested these for effects on the ability of cells to act as ICE*Bs1* recipients during conjugation. We found that an insertion of *spc* into a deletion of *yonF* (Δ*yonF*::*spc*) reduced acquisition of ICE*Bs1* only when *spc* was co-directional with *yonE*. Furthermore, deletion of *yonE* in this context eliminated the defect in acquisition of ICE*Bs1* (Fig 4B). In the absence of all other SPß genes, expression of *yonE* from the IPTG-inducible promoter Pspank(hy) was sufficient to inhibit acquisition of ICE*Bs1* (Fig 4B). We conclude that *yonE* in SPß is both necessary and sufficient to cause the decrease in stable acquisition of ICE*Bs1*. Results presented below demonstrate that when ICE*Bs1* is transferred to cells expressing *yonE*, those nascent transconjugants die. It is for this reason that there are no stable transconjugants recovered.

The decreased acquisition of ICE*Bs1* by recipients expressing *yonE* was dependent on the ICE*Bs1* gene *spbK*. We tested strains expressing *yonE* for the ability to acquire ICE*Bs1* that was missing *spbK* (ICE*Bs1* Δ*spbK*), and found that they all acquired the Δ*spbK* element at the same frequency as wild type recipients not expressing *yonE* (Fig 4B, right end of panel). From these results we conclude that expression of *yonE* caused a defect in the stable acquisition of ICE*Bs1* and that this defect was dependent on the presence of *spbK* in the incoming ICE*Bs1*. We note that loss of *spbK* caused no reduction in conjugation efficiency (Fig 4B), demonstrating that it is dispensable for conjugation. We also note that the presence or absence of wild type SPß had no detectable effect on conjugation efficiency (Fig 4B).

### Co-expression of *yonE* and *spbK* causes a defect in cell growth and a drop in cell viability

**Fig 5:**
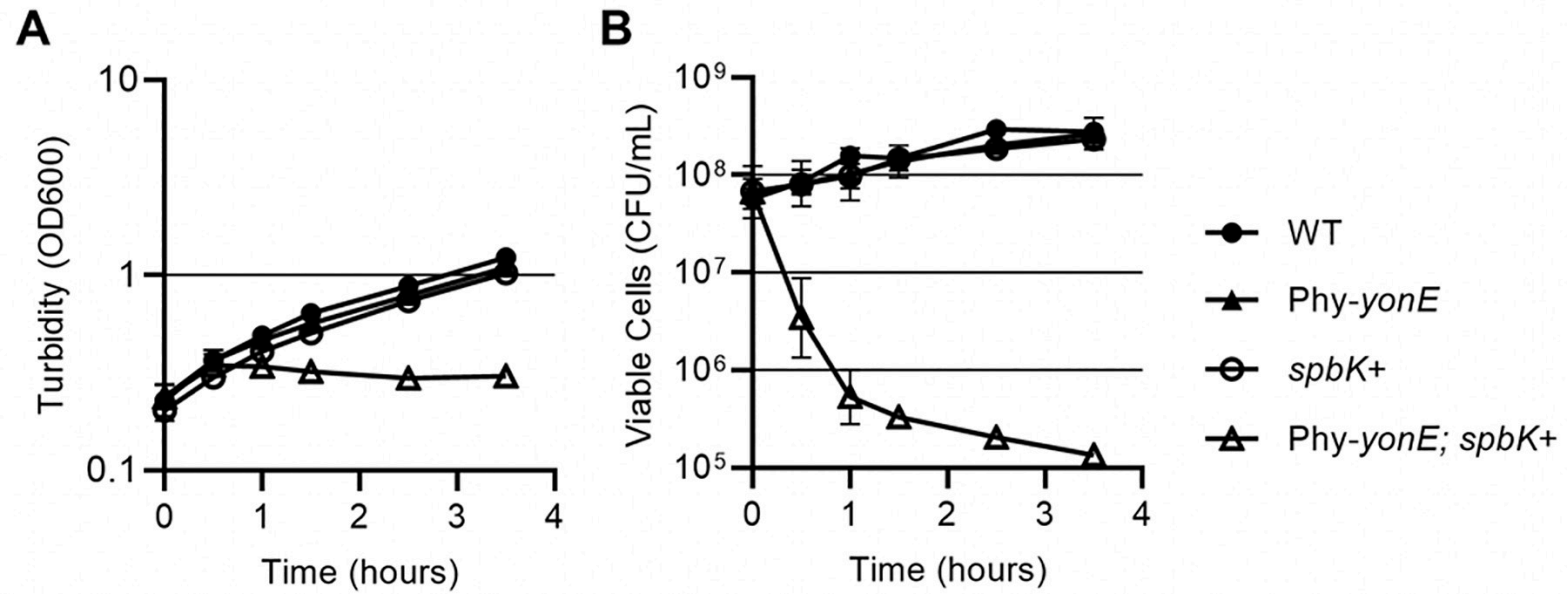
Co-expression of *spbK* and *yonE* kills cells and results in a growth defect. Strains null for ICE*Bs1* and SPß (closed circles, PY79), expressing *yonE* (closed triangles, *amyE*::{Pspank(hy)-*yonE*}, CMJ616), expressing *spbK* (open circles, *lacA*::*spbK*, CMJ684), or both *yonE* and *spbK* (open triangles, CMJ685) were grown in minimal medium. *yonE* expression was induced by the addition of 1 mM IPTG and the culture turbidity (**A**), and cell viability (**B**) were followed over time. T = 0 samples were collected immediately prior to induction with IPTG. Cell viability was measured as the number of colony forming units per ml, measured by plating for CFUs on LB plates made with Noble agar. Experiment was repeated 3 times.

We found that expression of *spbK* (from its own promoter) and *yonE* (from Pspank(hy)) together caused a severe growth defect. We grew cells containing both *spbK* and *yonE* in defined minimal medium and added IPTG (time = 0) to increase expression of *yonE* (Fig 5). This caused a rapid growth arrest as measured by optical density (Fig 5A) and an ~1000-fold drop in viability as measured by plating for CFUs on LB plates made with Noble agar (Fig 5B; see below). In contrast, expression of either gene alone, *spbK* from its own promoter (*lacA*::*spbK*), or *yonE* from an inducible promoter (*amyE*::Pspank(hy)-*yonE*), had no obvious effect on growth (Fig 5A and 5B). Together, our results indicate that co-expression of *yonE* and *spbK* is detrimental to cell growth. In an SPß lysogen that also contains ICE*Bs1*, *spbK*, is expressed, but *yonE* is not. *yonE* would be expressed only if SPß were activated, or upon infection of non-lysogens.

Despite growing normally in defined liquid medium prior to adding IPTG, cells containing both *lacA*::*spbK* and *amyE*::Pspank(hy)-*yonE* had a substantial plating defect (~200-fold) when plated on LB plates made with standard bacto-agar (Difco), and had a distinct small colony morphology even in the absence of IPTG. The plating and colony size defects were eliminated when the cells were plated on LB plates made with Noble agar (Difco) (*S1 Fig*), a purified form of agar that is used to culture some fastidious organisms. We hypothesize that a component of bacto-agar sensitizes cells to the detrimental impact of co-expressing *spbK* and *yonE*, such that leaky expression from Pspank(hy)-*yonE* is sufficient to trigger the growth defect.

### *yonE* is needed for phage production

**Fig 6:**
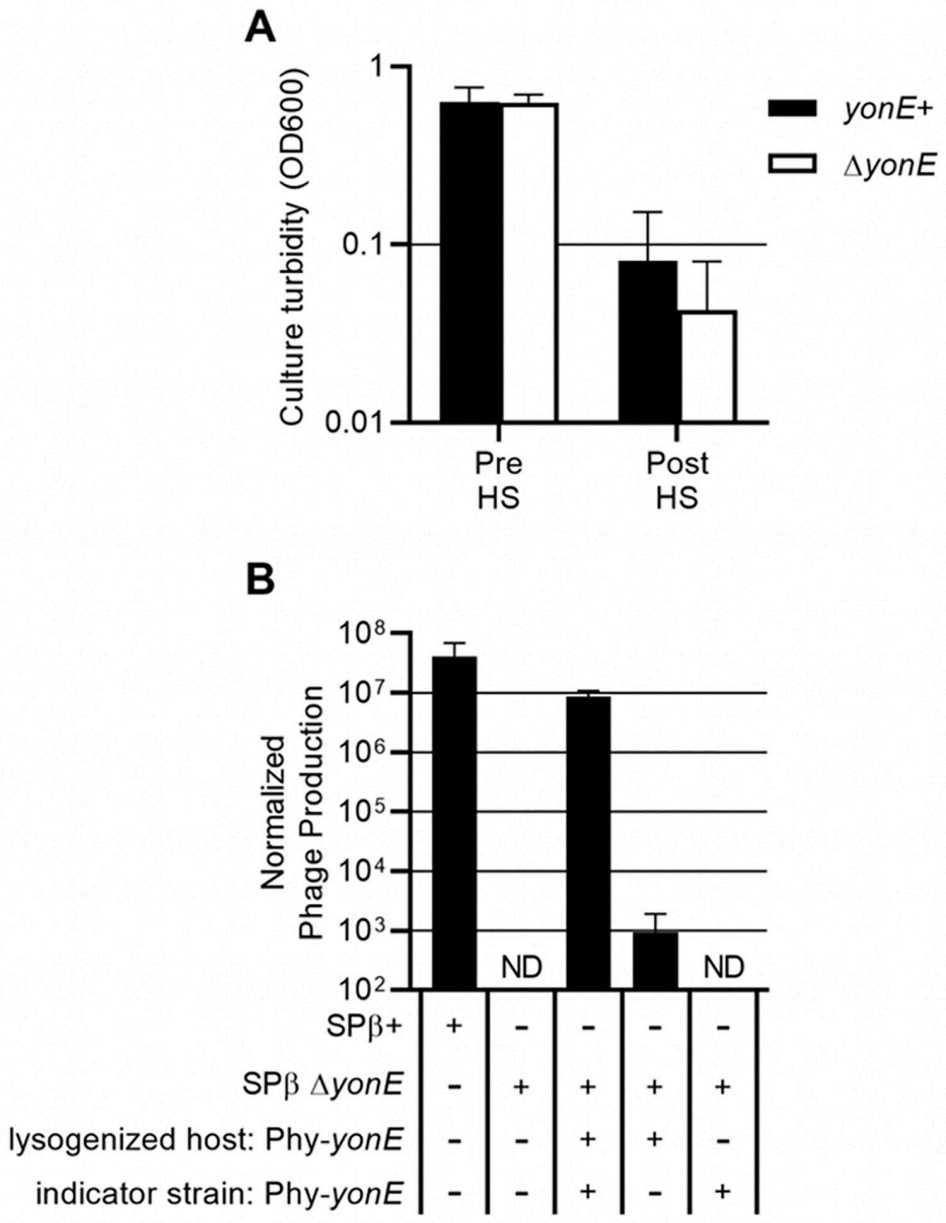
*yonE* is needed for production of infectious phage. **A.** Strains carrying a temperature-sensitive SPß lysogen with a wild type *yonE* allele (black bars, CMJ114) or Δ*yonE* (white bars, CMJ455) were cultured in rich medium, then heat shocked for 20 minutes (methods). The Y axis shows the average and standard deviation of the OD600 from each culture immediately before and 70 minutes (± 5 minutes) after the heat shock. Each experiment was repeated ≥3 times. **B.** Phage were prepared by culturing strains with a temperature sensitive SPß lysogen with a wild type *yonE* allele (CMJ114), Δ*yonE* (CMJ455), or a Δ*yonE* allele with *yonE* complemented from the chromosome (*lacA*::{Pspank(hy)-*yonE*}, CMJ457) to an OD600 of approximately 0.4, and then heat shocking the cultures and collecting phage (methods). Lysates were then spread on lawns of the indicator strain CU1050 or an indicator with *lacA*::{Pspank(hy)-*yonE*} (CMJ440) and incubated overnight to allow plaque formation. The Y-axis shows the average and standard deviation of the number of infectious phage /ml, normalized by dividing by the OD600 of the culture at the time of heat shock. ND = not detected. Each experiment was repeated ≥3 times.

To determine the effect of *yonE* on phage production, we made an unmarked deletion of *yonE* (Δ*yonE443*) in a temperature-sensitive SPß lysogen. We found that cultures of this inducible Δ*yonE* lysogen cleared comparably to a *yonE*+ strain following a shift to high temperature (Fig 6A), demonstrating that *yonE* is not needed for induction of SPß from a lysogenic state, nor is it needed to cause host cell lysis.

Despite the fact that the Δ*yonE* host cells lysed, there were no detectable viable phage (< 10 PFUs/ml) produced by the mutant lysogen (Fig 6B, first two columns). This defect in phage production was partially complemented by expression of *yonE* from an ectopic chromosomal locus. These Δ*yonE* phage (recovered from the complemented strain) were capable of forming plaques on an indicator strain that also expressed *yonE* (Fig 6B, last three columns). Although a small number of phage produced by a Δ*yonE* lysogen were able to form plaques on a *yonE*- indicator strain, analysis of lysogenized cells obtained from these plaques revealed that the phage had a wild type copy of *yonE*, likely obtained through homologous recombination with the *yonE* allele on the chromosome of the original host strain. Based on these results, we conclude that *yonE* is essential for production of SPß.

To determine if *yonE* is needed to form a lysogen, we made a stock of *spc*-marked Δ*yonE* phage by growing the Δ*yonE* mutant on a *B*. *subtilis* strain ectopically expressing *yonE*. The frequency of lysogenization of *spc*-marked *yonE*+ and Δ*yonE* phage were both approximately 1%, indicating that *yonE* is not needed for lysogen formation.

The function of *yonE* is not known. However, there are homologs in other phages, including the phage C-ST from *Clostridium botulinum*. The region of homology between C-ST and SPß extends from *yonG* to *yomZ*, indicating that these genes may encode conserved phage functions [43]. The phage E3 from *Geobacillus* encodes a putative portal protein (accession number AJA41333) that is 25% identical to YonE [44]. Portal proteins are one of three molecular components involved in packaging the phage genome into the capsid during maturation. The other two components are the large and small terminase subunits [45]. Additional homology searches using NCBI BLAST revealed that YonF is a member of the terminase 1 superfamily and encodes a terminase 6 multidomain, typical of large terminase subunit proteins. These results indicate that YonE and YonF may be a part of the SPß head packaging machinery. This notion is consistent with the need for *yonE* in production of functional phage, but not in host cell lysis or formation of lysogens.

### SpbK contains a TIR domain involved in protein-protein interaction

*spbK* is predicted to encode a 266 amino acid protein. Using the NCBI Delta-BLAST search tool [46] we found that the C-terminal region of SpbK (amino acids 113–266) contains a Toll Interleukin-1 Receptor (TIR) domain in the TIR_2 superfamily (accession: cl23749) (*S2A Fig*). Proteins containing TIR domains have been found in animals, plants, and bacteria. In animals, such proteins are involved in signaling cascades in development and in immune activation [47]. In plants they mediate disease resistance, often in response to infectious agents [48]. Some pathogenic bacteria encode TIR domain proteins that interact with eukaryotic host TIR domain proteins to modulate the host immune response [49]. Many non-pathogenic bacteria also contain TIR domain proteins and it is thought that the TIR domains mediate protein-protein interaction [50]. Recent work has also implicated some bacterial TIR domain proteins as components of anti-phage defense systems, though the mechanism of defense is not understood [51].

Where they have been studied, TIR domains mediate protein-protein interactions by interacting with other TIR domains. *spbK* is the only gene in *B*. *subtilis*, including all horizontally acquired sequences (e.g, ICE*Bs1* and SPß), predicted to encode a TIR domain. Using a yeast two-hybrid assay (Methods), we found that full-length SpbK multimerizes *in vivo* (*S2B Fig*). Additionally, we found that the TIR domain alone interacted with both full-length SpbK and with the TIR domain, but that deleting the TIR domain abolished all interaction between SbpK proteins (*S2B Fig*). We also tested for, but were unable to detect, interaction between SpbK and YonE, indicating that if these two proteins interact, that interaction was not detectable with the yeast two-hybrid system that we used (Methods).

### ICE*Bs1* protects *B*. *subtilis* populations from attack by SPß

**Fig 7:**
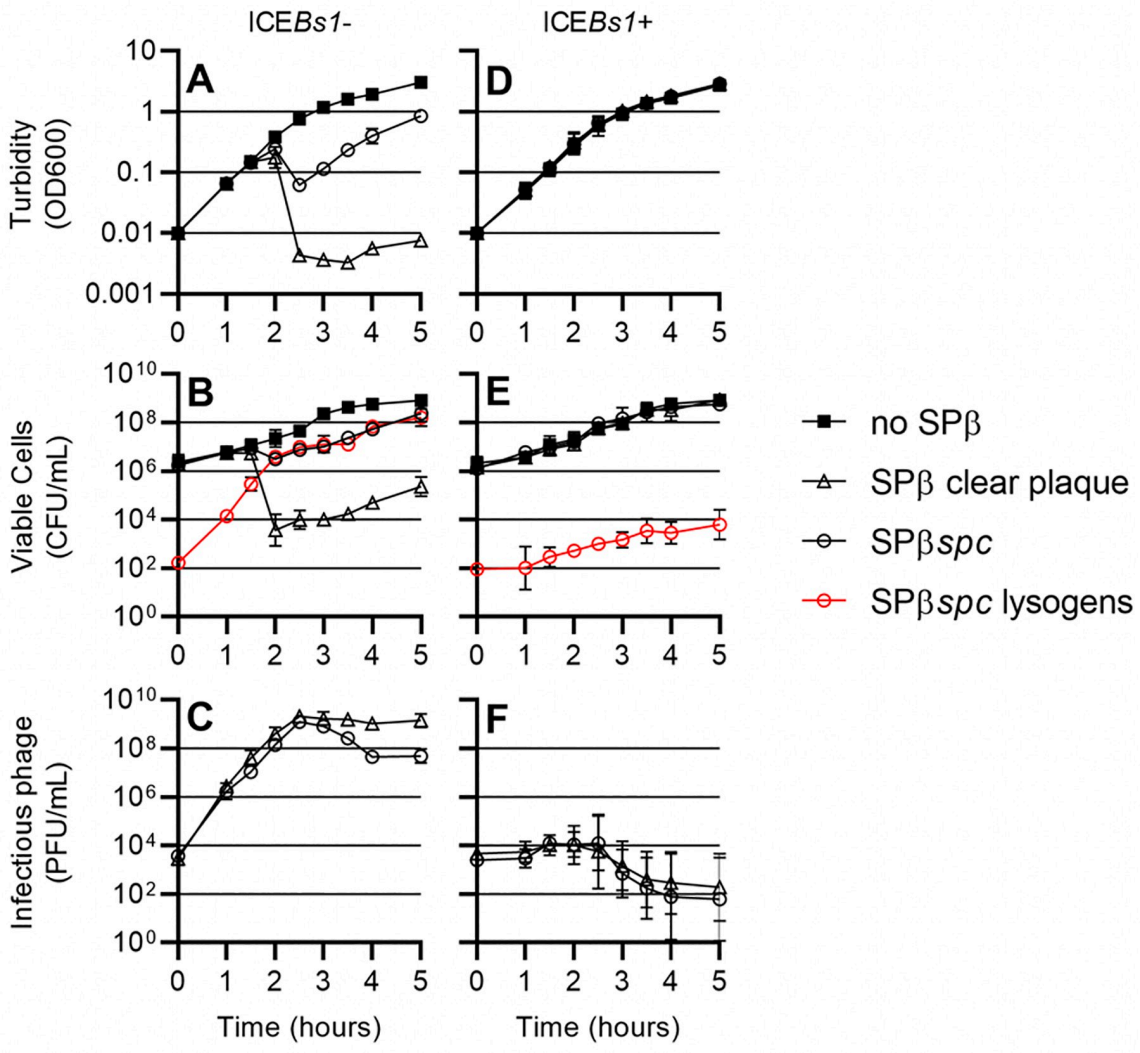
ICE*Bs1* protects *B*. *subtilis* populations against SPß. **A, B, C,** SPß^0^ ICE*Bs1*^0^ (CU1050) and **D, E, F,** SPß^0^ ICE*Bs1+* (CMJ81) were grown in rich medium, infected with no phage (filled squares), SPß clear plaque (open triangles), or SPß::*spc* (open circles) at an MOI of approximately 1:100 and diluted in fresh medium to an OD600 of 0.01. Culture turbidity (**A, D**), CFUs/ml (**B, E)**, and the number of infectious phage per ml of culture (**C, D**) were tracked over time. Additionally, for cultures infected with SPß::*spc*, the number of lysogenized cells were tracked over time (red open circles).

As described above, when SPß undergoes lysogenic to lytic conversion, SPß lysogens that contain ICE*Bs1* die without significant production of progeny phage. De novo infection of non-lysogens that contain ICE*Bs1* also die without producing progeny phage. We found that in a population of cells, this abortive infection system in ICE*Bs1* protected cells from killing by SPß. We grew SPß-cured strains of *B*. *subtilis* that either contained or did not contain ICE*Bs1*, infected the cultures with SPß at a low multiplicity of infection (MOI ~0.01), and tracked the growth (optical density) of the culture, the concentration of viable cells (including lysogens), and free phage over time (Fig 7). We suspected that use of SPß that is capable of making lysogens could mask possible effects on cell death and would be measuring possible protection of the population by ICE*Bs1* and by the formation of lysogens (which are themselves immune to superinfection, see below). Therefore, we first analyzed a clear plaque mutant (incapable of making lysogens; Methods) to eliminate possible effects of lysogeny. We then measured effects of ICE*Bs1* on phage that could form lysogens.

When cultures of an ICE*Bs1*^0^ strain were infected with a clear plaque mutant of SPß (MOI ~0.01) the cells continued to grow at the same rate as an uninfected culture for approximately 1.5 hours, then the majority of the cells abruptly died, as evidenced by a decrease in optical density (Fig 7A) and an approximately 5,000-fold decrease in CFUs (Fig 7B). During this time (1.5 hrs) the concentration of phage in the culture (Fig 7C) surpassed the concentration of cells (Fig 7B).

In contrast, when an ICE*Bs1*+ strain was infected with the clear plaque mutant of SPß (MOI ~0.01), cell growth was indistinguishable from an uninfected culture as measured by both the optical density (Fig 7D) and the number of CFUs (Fig 7E). The population of phage in the culture generally decreased to below the initial inoculum (Fig 7F). These results indicate that the presence of ICE*Bs1* is beneficial to the population of cells even though individual infected cells may not survive.

Experiments described above were done with an SPß mutant that was unable to form lysogens. We repeated these experiments using SPß::*spc*, that, other than the *spc* insertion is wild type and able to form lysogens. Lysogens were detected as spectinomycin-resistant colonies.

When cells without ICE*Bs1* were infected with SPß::*spc* (MOI ~0.01), there was a 10-fold drop in both the optical density of the culture (Fig 7A) and the number of CFUs (Fig 7B). During the experiment, many cells became lysogenized with SPß. Lysogens are then protected from killing by new SPß infection [24]. These lysogens continued to grow, and after about five hours the population of cells had increased and virtually all cells were SPß lysogens (Fig 7B). Although wt SPß killed only ~90% of the cells, (compared to >99.9% killing by the clear plaque mutant), wt SPß became established in the entire outgrown population.

When cells with ICE*Bs1* were infected with SPß::*spc* (MOI ~0.01), cell growth continued and there was no obvious drop in optical density (Fig 7D) nor in the number of CFUs (Fig 7E). Five hours after the initial infection, the number of phage in the culture was below the initial inoculum (Fig 7E) and the number of SPß lysogens remained at approximately 10^4^–10^5^ per ml (Fig 7F), a relatively small fraction of the total number of cells.

Together, these results indicate that the presence of ICE*Bs1* in cells limits phage production, thereby protecting a population of cells from predation by SPß. The presence of ICE*Bs1* does not limit initial lysogenization. However, because ICE*Bs1* limits the production of new phage, the number of lysogens is limited by the initial inoculum of phage. In this way, ICE*Bs1* protects the population from killing by SPß, and secondarily, prevents SPß from invading all the cells, thereby preventing lysogens taking over the population.

## Discussion

Results presented here demonstrate that ICE*Bs1* encodes an anti-phage system that inhibits production of the phage SPß. This inhibition occurs upon *de novo* infection by SPß and also upon induction of an SPß lysogen. There is little or no direct effect on the formation of lysogens. The ICE*Bs1* gene *spbK* is both necessary and sufficient to inhibit SPß: deleting *spbK* from ICE*Bs1* abolishes the phenotype, and expressing *spbK* in a strain missing ICE*Bs1* fully inhibits SPß. Expression of *yonE* apparently triggers this anti-phage response, and co-expression of *spbK* and *yonE* in a strain that otherwise lacks ICE*Bs1* and SPß rapidly kills the cells. There are multiple possibilities for how YonE triggers this killing. It could directly interact with SpbK and the two proteins together, perhaps with other host products, and could disrupt an essential host function. YonE could somehow modify SpbK, perhaps covalently, or by stabilizing it, thereby activating it. Alternatively, YonE could modify an essential host product, making cells susceptible to SpbK. Of course, it is possible that SpbK makes cells susceptible to YonE, and that cells with ICE*Bs1* are ’primed’ to be killed when *yonE* is expressed.

Whatever the mechanism, we conclude that ICE*Bs1* encodes an abortive infection system that protects its host from predation by SPß. Below, we briefly describe the *yonE* and *spbK* gene products and TIR-domains, comment generally on abortive infection systems, and conclude with general thoughts about cargo genes in ICEs.

### Genes involved in protection against SPß

*yonE* is essential for the phage lytic cycle, but is not required for lysogen formation. Bioinformatic analysis of *yonE* and *yonF* revealed a possible role for these genes as components of the phage head-packing machinery, needed during the final stages of a phage’s lytic cycle.

SpbK contains a TIR domain. Where TIR domains have been studied they generally mediate protein-protein interactions through recognition of other TIR domains. However, examples of heterotypic interactions of TIR domains with non-TIR domain proteins have been described [52]. In some bacterial pathogens, TIR-domain proteins modulate the host immune response [50,53–55]. Recent work has implicated some bacterial TIR-domain proteins as being essential components of a class of anti-phage defense systems (“Thoeris”) [51]. Two of these Thoeris systems (from *Bacillus cereus* and *Bacillus amyloliquefaciens*) have been shown to non-specifically confer resistance to some myophages when reconstituted in *B*. *subtilis*, although the mechanism of this resistance is not understood.

Although SpbK and the Thoeris systems appear to have a common purpose, SpbK does not appear to be a component of a *B*. *subtilis* Thoeris antiphage system. The hallmark of Thoeris systems is a single gene encoding a NAD+ binding protein (ThsA) in proximity to (typically multiple) genes encoding TIR-domain proteins (ThsB) [51]. *spbK* is the only gene encoding a TIR-domain protein in *B*. *subtilis*. Furthermore, of all the genes in ICE*Bs1*, *spbK* is both necessary and sufficient for protection from SPß, and there is no indication that SpbK contains a nucleotide binding domain. Irrespective of these differences, our analysis of SpbK raises the possibility that Thoeris anti-phage systems might function as abortive infection systems.

Many isolates of *B*. *subtilis* have both ICE*Bs1* and SPß. These elements likely co-evolved and it is possible that the *spbK*-mediated abortive infection is specific to SPß. However, we suspect that *spbK*-mediated abortive infection might respond to other phages, perhaps those with *yonE* orthologs.

### Abortive infection systems

The anti-phage phenotype encoded by ICE*Bs1* resembles abortive infection systems that have been described for *Lactococcus*, *Escherichia coli*, and other bacteria. Such systems detect infection of a bacterial cell by phage and respond by inhibiting a host process needed for phage maturation and release [56]. The mechanisms of inhibition vary widely, but often target a critical host process. The cellular process that is inhibited by SpbK is evidently essential for the host, as co-expression of *spbK* and *yonE* results in cell death. We have not yet determined what essential process(es) are targeted to cause SpbK-YonE-induced cell death.

### Cargo genes in ICEs

The cargo genes of mobile genetic elements, including ICEs, are those genes that are not necessary for the function of the mobile element, but are part of and transferred with the element. Cargo genes on a mobile element can often allow bacteria to rapidly acquire a new phenotype through acquisition of the element. Historically, most well studied ICEs were identified because of the phenotype(s) conferred by the cargo genes [6]. Identification and characterization of the responsible genes revealed that they were in an ICE.

Many ICEs are being identified by bioinformatic analyses of sequenced bacterial genomes [29,57]. In most of these analyses, it is not clear what, if any, phenotype is conferred by the ICE to its host. We suspect that many other ICEs with cargo genes of unknown function likely assist their hosts in mediating interactions with other mobile genetic elements.

## Methods

### Media and growth conditions

*E*. *coli* cells were grown in LB medium and on LB plates containing 1.5% agar at 37°C. *S*. *cereviseae* cells were grown in YPAD and on YPAD plates or synthetic dropout (SD) plates containing 2% agar and appropriate supplements to test for the indicated auxotrophies [58,59]. *B*. *subtilis* cells were grown in LB or S7_50_ defined minimal medium with 0.1% glutamate [60] with either glucose or arabinose (1% w/v) as a carbon source and on LB plates containing 1.5% bacto-agar or on Noble Agar (1.5%) for strains expressing both *spbK* and *yonE*. Antibiotics and other additives were used at the following concentrations for *E*. *coli*: carbenicillin (100 μg/ml), *B*. *subtilis*: kanamycin (5 μg/ml), spectinomycin (100 μg/ml), chloramphenicol (5 μg/ml). The Pspank(hy) promoter was activated with 1 mM isopropyl-ß-D-thiogalactopyranoside (IPTG), and the Pxyl promoter was activated with 1% (w/v) xylose, typically in cells grown in arabinose.

### Strains and alleles

*B*. *subtilis* strains and genotypes are listed in Table 1. Specific alleles are described below. All *B*. *subtilis* strains are derived from parent AG174 (JH642), unless otherwise indicated.

**Table 1:** *B*. *subtilis* strains used.

*B*. *subtilis* Strain	Genotype (reference)
AG174	*trpC2 pheA1* a.k.a., JH642 [27]
CAL321	*trpC2 pheA1* Δ(*rapI-yddM*)*318*::*kan* [39]
CAL322	*trpC2 pheA1* Δ(*yddG-yddM*)*319*::*kan* [39]
CAL323	*trpC2 pheA1* Δ(*yddB-yddM*)*320*::*kan* [39]
CAL347	*trpC2 pheA1* Δ(*ydcR-yddM*)*347*::*kan* [39]
CAL348	*trpC2 pheA1* Δ(*ydcQ-yddM*)*348*::*kan* [39]
CAL1500	*trpC2 pheA1* Δ(*spbK-rapI-phrI*)*1500*::*kan*
CMJ48	PY79 (ICE*Bs1*^0^) SPß+ *amyE*::{Pspank(hy)-empty *lacI spc*}
CMJ74	*trpC2 pheA1* ICE*Bs1*^0^ *amyE*::{*spbK cat*}
CMJ81	CU1050 (SPß^0^) ICE*Bs1*::*kan* (non-disruptive); note: made by crossing donor JMA448 with recipient CU1050
CMJ82	CU1050 (ICE*Bs1*^0^) (SPß^0^) *amyE*::{*spbK cat*}
CMJ98	*trpC2 pheA1* ICE*Bs1*^0^ *yolBC98*::*spc*
CMJ104	PY79 (ICE*Bs1*^0^) SPß+ Δ*yonF67*::*spc* [42]
CMJ114	CU1050 (ICE*Bs1*^0^) SPßc2 *yolBC98*::*spc*
CMJ116	CU1050 (ICE*Bs1*^0^) SPßc2 *yolBC98*::*spc amyE*::{*spbK cat*}
CMJ388	PY79 (ICE*Bs1*^0^) SPß+ Δ*yonF155*::*spc* (reverse orientation) [42]
CMJ402	PY79 (ICE*Bs1*^0^) SPß+ Δ*yonEF396*::*spc*
CMJ403	PY79 (ICE*Bs1*^0^) (SPß^0^) *amyE*::{Pspank(hy)-empty *lacI spc*} *lacA*::{Pspank(hy)-*yonE lacI mls*}
CMJ405	PY79 (ICE*Bs1*^0^) (SPß^0^) *amyE*::{Pspank(hy)-empty *lacI spc*} *lacA*::{Pspank(hy)-empty *lacI mls*}
CMJ431	*trpC2 pheA1 amyE*::{Pxyl-*rapI xylR cat*} Δ(*spbK-rapI-phrI*)*1500*::*kan*
CMJ434	*trpC2 pheA1 argA85*::Tn*917* SPßc2 Δ*yonE434*::*lox*-*cat*
CMJ440	CU1050 (ICE*Bs1*^0^) (SPß^0^) *lacA*::{Pspank(hy)-*yonE lacI mls*}
CMJ443	CU1050 (ICE*Bs1*^0^) SPßc2 Δ*yonE443* (unmarked)
CMJ455	CU1050 (ICE*Bs1*^0^) SPßc2 Δ*yonE443* (unmarked) *yolBC98*::*spc*
CMJ457	CU1050 (ICE*Bs1*^0^) SPßc2 Δ*yonE443* (unmarked) *yolBC98*::*spc lacA*::{Pspank(hy)-*yonE lacI mls*}
CMJ472	CU1050 (ICE*Bs1*^0^) (SPß^0^) *comK*::*cat*
CMJ616	PY79 (ICE*Bs1*^0^) (SPß^0^) *amyE*::{Pspank(hy)-*yonE lacI spc*}
CMJ684	PY79 (ICE*Bs1*^0^) (SPß^0^) *lacA*::{*spbK kan*}
CMJ685	PY79 (ICE*Bs1*^0^) (SPß^0^) *amyE*::{Pspank(hy)-*yonE lacI spc*} *lacA*::{*spbK kan*}
CMJ826	CU1050 SPßc2 *yolBC98*::*spc* ICE*Bs1*::*kan* (non-disruptive)
CMJ827	CU1050 (SPß^0^) ICE*Bs1*::*kan*, *comK*::*cat*
CMJ913	CU1050 (SPß^0^) ICE*Bs1*+ Δ(*rapI-phrI*)*342*::*kan*
CMJ914	CU1050 (SPß^0^) ICE*Bs1*+ Δ(*spbK-rapI-phrI*)*1500*::*kan*
CMJ917	CU1050 SPßc2 ICE*Bs1*+ Δ(*rapI-phrI*)*342*::*kan*, *yolBC98*::*spc*
CMJ918	CU1050 SPßc2 ICE*Bs1*+ Δ(*spbK-rapI-phrI*)*1500*::*kan*, *yolBC98*::*spc*
CU1050	ICE*Bs1*^0^ SPß^0^ *metA thrC leu codY sup-3* (*trnS-lys*) [31,33]; (CMJ28)
IRN342	*trpC2 pheA1* Δ(*rapI-phrI*)*342*::*kan* [22]
JMA222	*trpC2 pheA1* ICE*Bs1*^0^ [22]
JMA448	*trpC2 pheA1* ICE*Bs1*::*kan amyE*::{Pspank(hy)-*rapI spc*} [22] note: used as donor with recipient CU1050 to create CMJ81
KM250	*trpC2 pheA1* Δ(*rapI-phrI*)*342*::*kan amyE*::{Pxyl-*rapI cat*} [61]
PY79	ICE*Bs1*^0^ SPß^0^ [62]

#### SPß::*spc*

*spc* (spectinomycin resistance) was inserted between *yolB* and *yolC* in SPß. *spc* was amplified by PCR, and Gibson assembly [63] was used to join this fragment to genomic sequences containing the apparent bidirectional terminator located between the convergently transcribed genes *yolB* and *yolC* [64] such that a copy of the terminator is located on each side of *spc*. This was then used to transform naturally competent *B*. *subtilis* cells selecting for resistance to spectinomycin. An antibiotic resistant strain (CMJ98) was identified and the location of the *spc* gene verified by sequencing. This strategy resulted in duplication of the terminator with *spc* located between the terminators. The resulting phage is referred to as SPß::*spc98*, or SPß::*spc*.

#### Clear plaque mutant of SPß

SPß typically makes turbid plaques, characteristic of temperate phages. In the course of determining the titre of a stock of SPß::*spc*, we noticed a plaque that appeared clear. We picked this plaque and designated the phage SPß::*spc-clear*. We grew the phage and then tested for the ability to form lysogens by infecting cells and selecting for spectinomycin resistance. Cells infected with the SPß::*spc* phage readily formed lysogens. We did not detect any lysogens (spectinomycin resistance) from cells infected with SPß::*spc-clear*. In addition, all plaques observed were clear, verifying that this phage was indeed unable to form lysogens.

#### *Δ*(*spbK-rapI-phrI*)*1500*::*kan*

The region of ICE*Bs1* encoding *spbK*-*rapI*-*phrI* was replaced with *kan*. The deletion replaces all of *spbK*, *rapI*, and *phrI*, and was designed such that the orientation of *kan* and the *phrI* deletion boundary would be identical to the Δ(*rapI-phrI*)*342*::*kan* allele of IRN342 [22]. The linkage between *ΔspbK* and Δ(*rapI-phrI*)*342*::*kan* was used to transfer the *ΔspbK* allele as needed.

#### Δ*yonEF396*::*spc*

A deletion-insertion that replaces *yonE* and *yonF* with a co-directional *spc* insertion.

#### ΔyonE443

The unmarked Δ*yonE443* allele was made by replacing *yonE* with the *cat* gene, flanked by *lox* sites (CMJ434). The Cre recombinase, expressed from the temperature-sensitive plasmid, pDR244, was then used to remove the *lox*-flanked *cat* gene by recombination. Strains were then cured of pDR244 by culturing them on LB + 1.5% agar at 45°C, as previously described [42,65], resulting in strain CMJ443.

#### Overexpression of *yonE*

The *yonE* coding sequence was cloned into a plasmid containing the IPTG-inducible Pspank(hy) promoter [66], *lacI*, and either *spc* situated between genomic sequence from *amyE*, or *mls* situated between genomic sequence from *lacA*. The resulting construct was then transformed into competent *B*. *subtilis* cells. The following strains carrying a double-crossover of the given construct were identified by antibiotic resistance and PCR: CMJ403, *lacA*::{Pspank(hy)-*yonE lacI mls*}; CMJ616, *amyE*::{Pspank(hy)-*yonE lacI spc*}. Pspank(hy) is only partly repressed by LacI and was fully derepressed upon addition of 1 mM IPTG. Constructs lacking the *yonE* insert were also transformed into *B*. *subtilis* to generate the control alleles *lacA*::{Pspank(hy)-empty *lacI mls*} and *amyE*::{Pspank(hy)-empty *lacI spc*}.

#### Expression of *spbK*

To study expression of *spbK* in the absence of other ICE*Bs1* genes, a fragment containing the *spbK* coding sequence and 330 bp upstream was amplified by PCR and cloned into a plasmid for double-crossover integration into *lacA* or *amyE*. For cloning into *lacA*, the *spbK* fragment was cloned by Gibson assembly into a plasmid containing *kan* and parts of *lacA* suitable for double crossover. For cloning into *amyE*, the *spbK* fragment was cloned into a plasmid containing *cat* flanked by genomic sequences flanking the *amyE* locus by Gibson assembly. The resulting constructs were transformed into naturally competent *B*. *subtilis* cells and strains carrying a double crossover were identified as above, resulting in strains CMJ74 {*amyE*:: (*spbK cat*)} and CMJ684 {*lacA*:: (*spbK kan*)}.

### Plaque assays

To quantify the number of PFUs, samples with phage were diluted in LB and 100 μl of appropriate dilutions were mixed with 300 μl of an indicator strain at an OD600 of 0.5. Phage and cells were incubated at room temperature for 5 minutes, then mixed with 3 ml of soft agar (soft agar contains 10 g/l tryptone, 5 g/l yeast extract, 10 g/l NaCl, 6.5 g/l agar). The soft agar was spread on warm LB plates and incubated overnight at 37°C, allowing a lawn of cells to form. Plaques in the lawn were then counted. To photograph plaques, bacterial lawns were stained with 2,3,5—triphenyltetrazolium chloride [67] (TTC, Sigma). Briefly, 8 ml of 0.1% TTC in LB was pipetted onto plates and incubated at 37°C for 30 minutes. The TTC solution was then aspirated off and the petri dishes were photographed.

### Single-round infection experiments

Cells of the strain to be infected were cultured in rich medium to mid- to late exponential phase. The OD600 was then adjusted to 0.5 and 100 μl of cells was mixed with 10 μl medium containing 10^5^ PFU of SPß (MOI 1:100). Cells and phage were co-incubated for 5 min at 37°C, then washed 3 times by adding 1 ml LB, pelleting the cells in a microcentrifuge and removing the supernatant. The washed pellet was resuspended in 10 ml LB and incubated at 37°C with aeration to allow the phage to develop. Samples of the culture were taken at various time points and used immediately for plaque assays to quantify the concentration of infective centers (free phage + infected cells) in the culture.

### Quantification of lysogeny

The frequency of lysogenization was determined using SPß::*spc98*. 10^4^ PFUs of SPß::*spc98* in 10 μl LB were added to 100 μl of an indicator strain at an OD600 of 0.5 (an MOI of approximately 1:1000). Phage and cells were incubated at room temperature for 5 minutes, then cells were washed 3x with 1 ml LB to remove unbound phage. Cells were then spread on LB plates with spectinomycin to select for cells that had become lysongenized with SPß::*spc*.

### Yeast two-hybrid assays

Yeast strains are listed in Table 2 and were derived from PJ69-4A [59]. The yeast two hybrid strains and vectors used in this study have been previously described [59]. Briefly, the coding sequence for *spbK* from amino acids 1–104 (N-terminus), 97–266 (TIR domain) and full length *spbK* were cloned into pGAD-C1 and fused to the *GAL4* activation domain or pGBDU-C3 and fused to the *GAL4* DNA binding domain. These vectors were then transformed into competent PJ69-4A cells using the LiAc method of Gietz and Schiestl [58] and plated on synthetic dropout (SD) medium with appropriate supplements to select for acquisition of the plasmids. The ability to grow in the absence of leucine (pGAD-based plasmids) or uridine (pGBDU-based plasmids) was used to select clones that acquired each plasmid. To test for interaction between peptides, yeast strains carrying the plasmids of interest were spotted on SD medium and scored for growth in the absence of adenine, with growth indicating an interaction. As a control, strains carrying each individual plasmid were also scored for growth in the absence of adenine (all were negative).

**Table 2:** Yeast strains used.

yeast strain	genotype
CMJ620	*S*. *cereviseae* mat a ura3-52 leu2-3 his3 trp1 dph2Δ::HIS3 gal4Δ gal80Δ GAL2-ADE2 LYS2::GAL1-HIS3 met2::GAL-lacZ pCJ113 pCJ107
CMJ621	*S*. *cereviseae* mat a ura3-52 leu2-3 his3 trp1 dph2Δ::HIS3 gal4Δ gal80Δ GAL2-ADE2 LYS2::GAL1-HIS3 met2::GAL-lacZ pCJ113 pCJ108
CMJ622	*S*. *cereviseae* mat a ura3-52 leu2-3 his3 trp1 dph2Δ::HIS3 gal4Δ gal80Δ GAL2-ADE2 LYS2::GAL1-HIS3 met2::GAL-lacZ pCJ113 pCJ109
CMJ626	*S*. *cereviseae* mat a ura3-52 leu2-3 his3 trp1 dph2Δ::HIS3 gal4Δ gal80Δ GAL2-ADE2 LYS2::GAL1-HIS3 met2::GAL-lacZ pCJ114 pCJ107
CMJ627	*S*. *cereviseae* mat a ura3-52 leu2-3 his3 trp1 dph2Δ::HIS3 gal4Δ gal80Δ GAL2-ADE2 LYS2::GAL1-HIS3 met2::GAL-lacZ pCJ114 pCJ108
CMJ628	*S*. *cereviseae* mat a ura3-52 leu2-3 his3 trp1 dph2Δ::HIS3 gal4Δ gal80Δ GAL2-ADE2 LYS2::GAL1-HIS3 met2::GAL-lacZ pCJ114 pCJ109
CMJ632	*S*. *cereviseae* mat a ura3-52 leu2-3 his3 trp1 dph2Δ::HIS3 gal4Δ gal80Δ GAL2-ADE2 LYS2::GAL1-HIS3 met2::GAL-lacZ pCJ115 pCJ107
CMJ633	*S*. *cereviseae* mat a ura3-52 leu2-3 his3 trp1 dph2Δ::HIS3 gal4Δ gal80Δ GAL2-ADE2 LYS2::GAL1-HIS3 met2::GAL-lacZ pCJ115 pCJ108
CMJ634	*S*. *cereviseae* mat a ura3-52 leu2-3 his3 trp1 dph2Δ::HIS3 gal4Δ gal80Δ GAL2-ADE2 LYS2::GAL1-HIS3 met2::GAL-lacZ pCJ115 pCJ109

### Mating assays

Mating assays were performed as previously described [38,42]. Briefly, donors and recipients were grown separately in minimal medium with 1% arabinose as a carbon source. RapI expression was induced in donors for 2 hours with 1% xylose. Approximately equal numbers of donors and recipients were then mixed, collected on a filter and placed on 1.5% agar plates buffered with Spizizens minimal salts (SMS agar contains 15 mM ammonium sulfate, 80 mM dibasic potassium phosphate, 44 mM monobasic potassium phosphate, 3.4 mM trisodium citrate, 0.8 mM magnesium sulfate, and 1.5% agar at pH 7.0) [68] for 90 minutes. Cells were rinsed off the filter, diluted, and spread on LB plates with selective antibiotics and incubated at 37°C overnight before quantification of colony forming units.

## Supporting information

S1 Fig Co-expression of *spbK* and *yonE* results in a sensitivity to standard bacteriological agar. Strains null for ICE*Bs1* and SPß (PY79), expressing *yonE* (*amyE*::Phy-*yonE*, CMJ616), expressing *spbK* (*lacA*::*spbK*, CMJ684), or both *yonE* and *spbK* (CMJ685) were grown in minimal medium in the absence of IPTG. At an OD600 of 0.2, cultures were plated for CFUs on LB plates made with standard bacteriological agar (black bars) or on LB plates made with more rigorously purified Noble agar (white bars). Plating efficiency measured as CFUs/ml normalized to OD600.
(TIF)

S2 Fig SpbK contains a TIR domain that mediates self-interaction. **A.** Map of the SpbK peptide sequence showing the location of the TIR domain in black. **B.** Yeast two-hybrid screen of SpbK fragments. Yeast strains carrying full length SpbK (Full), SpbK amino acids 1–104 (N-term) or SpbK amino acids 97–266 (TIR) bound to the *GAL4* DNA binding domain (DBD, Y-axis) and/or the *GAL4* activation domain (AD, X-axis) were spotted on medium selective for interaction between the bait and prey peptides and incubated at 30° C to allow for growth (methods). The following combinations were tested: AD-SpbK + DBD-SpbK (CMJ620), AD-N-term + DBD-SpbK (CMJ621), AD-TIR + DBD-SpbK (CMJ622), AD-SpbK + DBD-N-term (CMJ626), AD-N-term + DBD- N-term (CMJ627), AD-TIR + DBD- N-term (CMJ628), AD-SpbK + DBD-TIR (CMJ632), AD-N-term + DBD-TIR (CMJ633), AD-TIR + DBD-TIR (CMJ634).
(TIF)
