# Supplementary figures and images for: Interactions between mobile genetic elements: An anti-phage gene in an integrative and conjugative element protects host cells from predation by a temperate bacteriophage
Source: PLoS Genet. 2022 Feb 14;18(2):e1010065. doi: 10.1371/journal.pgen.1010065 (PMC8880864; doi:10.1371/journal.pgen.1010065)

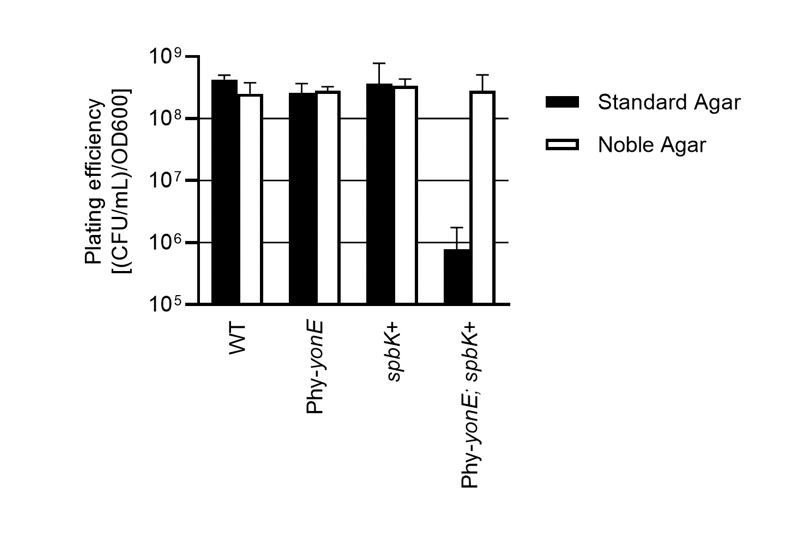

Supplement: S1 Fig — Strains null for ICEBs1 and SPß (PY79), expressing yonE (amyE::Phy-yonE, CMJ616), expressing spbK (lacA::spbK, CMJ684), or both yonE and spbK (CMJ685) were grown in minimal medium in the absence of IPTG. At an OD600 of 0.2, cultures were plated for CFUs on LB plates made with standard bacteriological agar (black bars) or on LB plates made with more rigorously purified Noble agar (white bars). Plating efficiency measured as CFUs/ml normalized to OD600. (TIF) [file pgen.1010065.s001.tif]

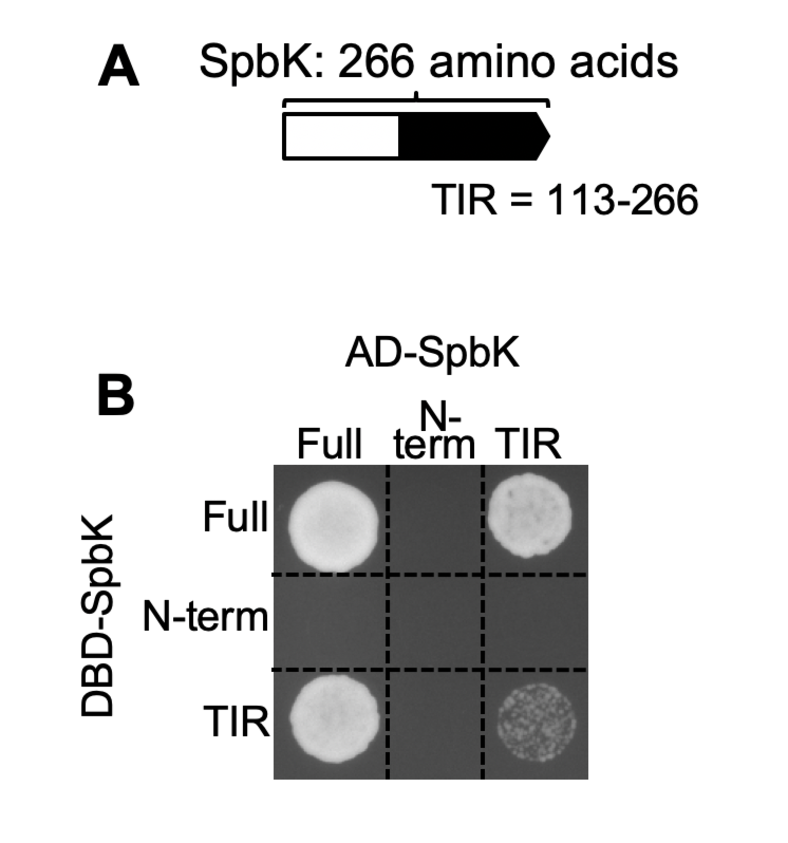

Supplement: S2 Fig — A. Map of the SpbK peptide sequence showing the location of the TIR domain in black. B. Yeast two-hybrid screen of SpbK fragments. Yeast strains carrying full length SpbK (Full), SpbK amino acids 1–104 (N-term) or SpbK amino acids 97–266 (TIR) bound to the GAL4 DNA binding domain (DBD, Y-axis) and/or the GAL4 activation domain (AD, X-axis) were spotted on medium selective for interaction between the bait and prey peptides and incubated at 30° C to allow for growth (methods). The following combinations were tested: AD-SpbK + DBD-SpbK (CMJ620), AD-N-term + DBD-SpbK (CMJ621), AD-TIR + DBD-SpbK (CMJ622), AD-SpbK + DBD-N-term (CMJ626), AD-N-term + DBD- N-term (CMJ627), AD-TIR + DBD- N-term (CMJ628), AD-SpbK + DBD-TIR (CMJ632), AD-N-term + DBD-TIR (CMJ633), AD-TIR + DBD-TIR (CMJ634). (TIF) [file pgen.1010065.s002.tif]
